# Supplementary figures and images for: Identification of physiological adverse events using continuous vital signs monitoring during paediatric critical care transport: A novel data-driven approach
Source: PLOS Digit Health. 2025 Sep 25;4(9):e0000822. doi: 10.1371/journal.pdig.0000822 (PMC12463272; doi:10.1371/journal.pdig.0000822)

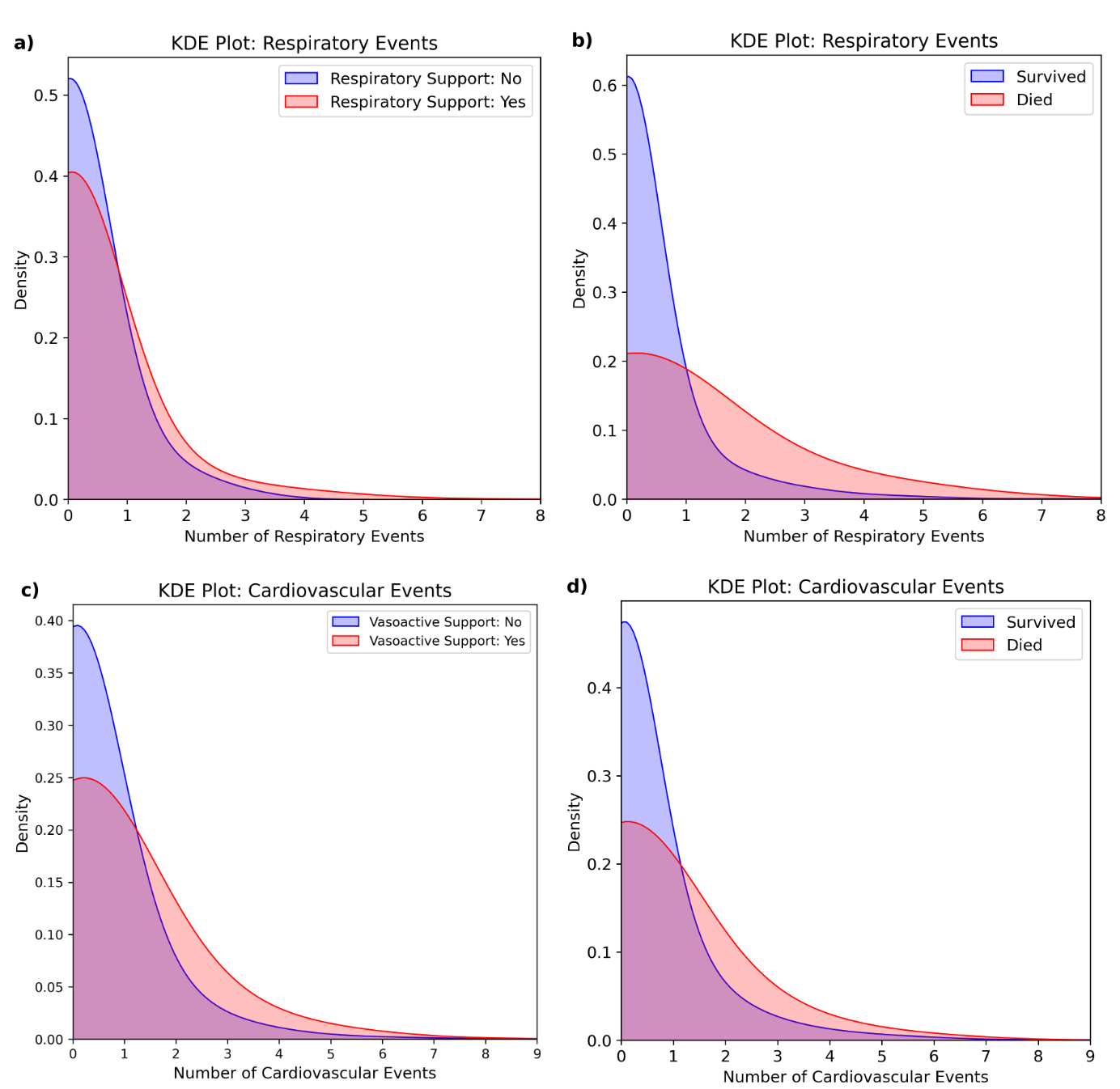

Supplement: S1 Fig — (b) KDE plot show that patients with more adverse respiratory events have higher risk of 30-day mortality. (c) KDE plot show that patients with more adverse cardiovascular events are more likely to receive cardiovascular support during transport. (d) KDE plot shows that patients with more adverse cardiovascular events have higher risk of 30-day mortality. (TIF) [file pdig.0000822.s001.tif]

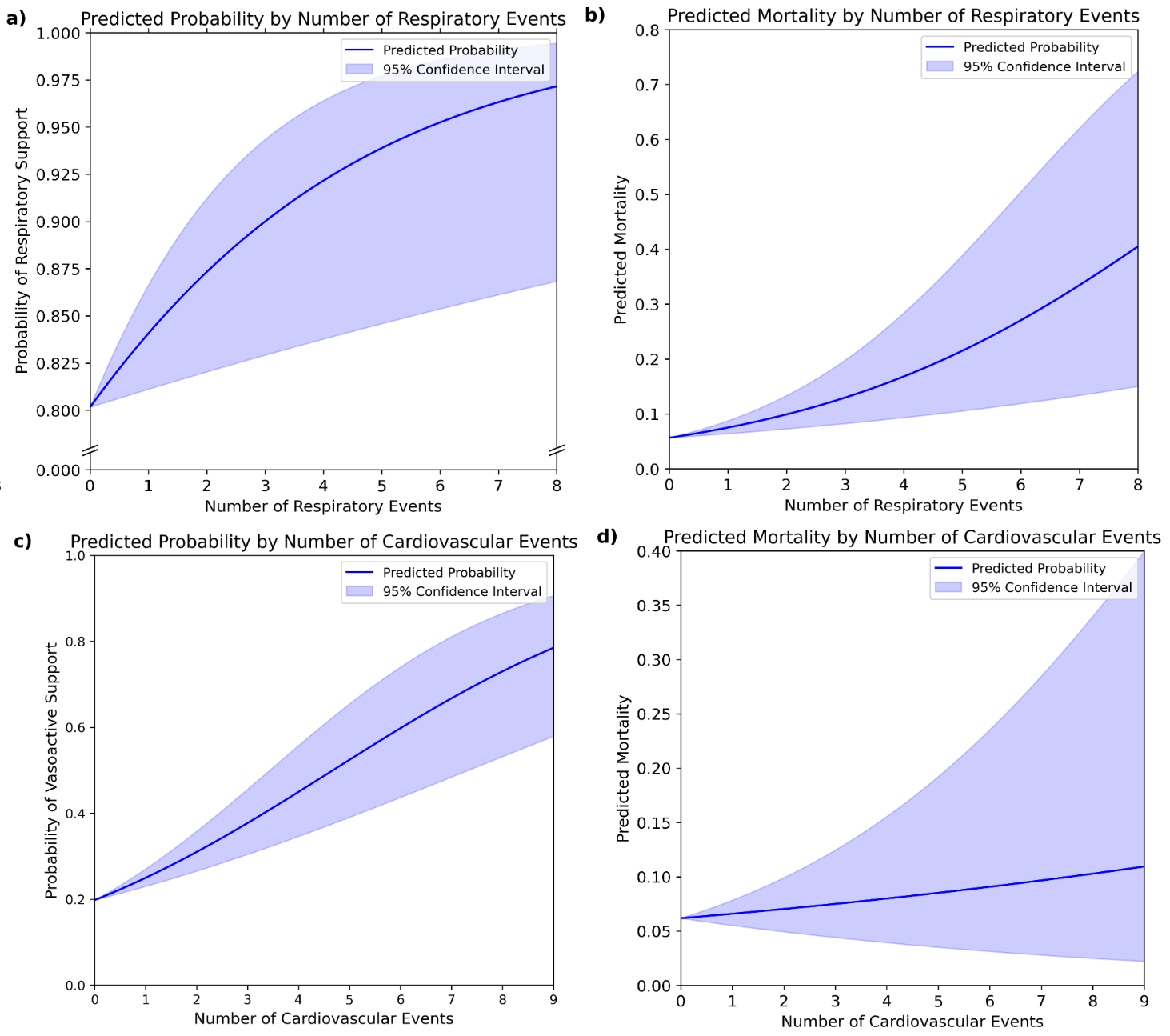

Supplement: S2 Fig — (c) Logistic regressions indicate that increasing numbers of adverse cardiovascular events are associated with increasing probability of receiving cardiovascular support and 30-day mortality, however the trend for 30-day mortality is not statistically significant at the 95% confidence level. (TIF) [file pdig.0000822.s002.tif]
