# Supplementary material for: Identification of physiological adverse events using continuous vital signs monitoring during paediatric critical care transport: A novel data-driven approach
Source: PLOS Digit Health. 2025 Sep 25;4(9):e0000822. doi: 10.1371/journal.pdig.0000822 (PMC12463272; doi:10.1371/journal.pdig.0000822)
Supplement: Attachment — Submitted filename: Response to reviewers 0.1.docx [file pdig.0000822.s003.docx]

## **Response to Reviewers: PDIG-D-25-00160**

**Title:** Identification of physiological adverse events using continuous vital signs monitoring during paediatric critical care transport: a novel data-driven approach

**Dear PLoS Digital Health team,**

Thank you for the opportunity to revise and resubmit our manuscript, PDIG-D-25-00160, titled "Identification of physiological adverse events using continuous vital signs monitoring during paediatric critical care transport: a novel data-driven approach". We appreciate the thorough and constructive feedback from the Section Editor and the reviewers, which has helped us improve our manuscript.

We have addressed all comments raised by the editor and reviewers point-by-point below. Changes in the manuscript are highlighted in the "Revised Manuscript with Track Chang**es"** file.

**Have the authors made all data underlying the findings in their manuscript fully available (please refer to the Data Availability Statement at the start of the manuscript PDF file)?**

*Response:* We note the disagreement between the two reviewers regarding data availability. We have updated our statement to be more clear as follows: “The patient physiological data underlying the findings of this study are third-party data subject to ethical and legal restrictions due to their sensitive patient information content. This dataset was obtained from routine clinical records held by the Children’s Acute Transport Service (CATS) and Great Ormond Street Hospital (GOSH). Our research use of this dataset was approved by GOSH Research and Innovation Division, under ethical approvals for use of routine de-identified healthcare and operational hospital data (Research Database, NHS REC reference 21/LO/0646). The authors did not receive any special privileges in accessing the data that other researchers would not have.

Researchers seeking access to this dataset for secondary analysis must obtain permission directly from the data custodians. Interested researchers should contact the Research and Innovation Division at Great Ormond Street Hospital for Children NHS Foundation Trust (Research.Governance@gosh.nhs.uk). Data access applications are subject to a formal review process, including confirmation of appropriate ethical approval and the establishment of a Data Sharing Agreement with the requesting institution.”

## **Reviewer #1**

- We appreciate Reviewer #1's positive feedback regarding the accessibility and coherence of our manuscript, and the novelty and practical application of our approach.
- **Reviewer Comment: “I'd be interested in to know if patients being transported, by definition having been stabilised to some extent, are different from an vital signs perspective from patients conveyed to hospital as an emergency presentation (i.e from home). This may cause this application of this approach to be relevant only to transfers (but I suspect it does have wider application).”**

*Response*: We appreciate the reviewer raising this interesting and clinically relevant point. We agree that, given the stabilization and ongoing interventions often occurring prior to inter-hospital transfer, the physiological profiles and vital sign patterns of transported critically ill children may indeed differ from those presenting directly to an Emergency Department from home.

Our cohort, for instance, largely comprises highly acute patients already receiving significant support. For example, 70.6% were invasively ventilated (allowing for EtCO2 monitoring), and an additional 8.5% received non-invasive ventilatory support. Furthermore, 32.1% were on some form of vasoactive infusion during transport (as detailed in Table 1). Critically, the continuous, high-resolution vital sign data available in our study, including EtCO2 and invasive arterial blood pressure (in 73% and 36% of transports, respectively, per Table 2), are typically only available in invasively monitored patients, which would be far less common in an emergency transport directly from home to the emergency department.

This high acuity and the detailed data captured highlight precisely why our personalized, data-driven approach is particularly valuable for this cohort. Traditional fixed thresholds, often derived from general paediatric populations or emergency presentations, are less likely to be appropriate for these complex and often unstable patients, whose 'normal' vital signs are profoundly impacted by their illness severity and ongoing intensive care. Our method's ability to dynamically establish patient-specific stable ranges and detect deviations from these individual baselines is designed to be highly adaptable to such varying and complex physiological states.

While our study focuses on the inter-hospital transport setting, as outlined already in our discussion we believe the fundamental principles of our methodology, particularly its ability to learn and adapt to an individual's evolving physiological baseline regardless of the available vital signs, are transferable to other clinical environments. This includes emergency transfers from home to hospital, as well as patients admitted to Intensive Care Units where high-frequency, detailed monitoring is the norm.

Further research would certainly be valuable to assess the performance of our approach across these distinct clinical settings.

- **Reviewer Comment “I did also wonder whether gradual trends of increase or decrease were associated with specific outcomes and wonder if this could be measured?”:**

*Response:* We thank the reviewer for this insightful question regarding the association of gradual trends with specific outcomes and their measurement. Our current approach, utilizing Bollinger Bands, is primarily designed to detect significant, acute deviations from a dynamically established personalized baseline, rather than to explicitly characterize or quantify gradual trends. As we discuss in the manuscript (specifically in the Discussion, under the paragraph beginning "Additionally, the emphasis of our methodology..."), our method allows gradual trends to be absorbed within the continuously adjusting dynamic stable ranges, as focusing on sharp deviations helps differentiate acute deteriorations from expected physiological evolution.

However, the concept of vital sign trajectories and their association with outcomes is an important and complementary area of research. Indeed, using this same dataset, our group has previously investigated the distribution and trajectory of vital signs during paediatric critical care transport (Huo et al., 2023), observing differences in the progression of vital signs between age groups, diagnosis groups, and severity of illness groups.

While our current methodology prioritizes the detection of acute events, we acknowledge that developing approaches specifically designed to identify and quantify the clinical significance of insidious, gradual declines or improvements in vital signs would be a valuable direction for future work. We have explicitly noted this limitation and potential future research avenue within the Discussion section of the manuscript.

## **Reviewer #2**

- **Reviewer Comment: “Thank you for this interesting study on detecting physiological adverse events from vital signs data during pediatric transport. I thought the associations between the detected events and short- and long-term outcomes were particularly interesting. However, I note that there was no comparator approach (such as fixed vital-sign cutoffs) against which to compare the novel approach which calculates personalised and time-varying normal ranges. Therefore, it was difficult to evaluate the potential benefit of the proposed approach beyond existing approaches.”:**

*Response:* We appreciate the reviewer's thoughtful comment and the suggestion regarding the inclusion of a comparator approach, such as fixed vital sign cutoffs. We acknowledge that directly demonstrating the benefit of a novel method through comparison is valuable. However, for critically ill children undergoing inter-hospital transport, the concept of universally applicable fixed vital sign cutoffs presents significant clinical challenges and is often not a feasible or appropriate comparator due to the inherent heterogeneity and complexity of this patient population.

Our primary rationale for developing a personalized, data-driven approach stems from the absence of "normal" vital sign ranges for critically unwell children in transport. Unlike healthy children, these patients often have individualized physiological targets that dynamically change based on their age, underlying pathology, severity of illness, and ongoing interventions. For example:

- End-tidal CO2 (EtCO2) targets vary significantly; we might aim for a lower EtCO2 (e.g., 4.5-5 kPa) for neuroprotection in a patient with traumatic brain injury, while tolerating permissive hypercapnia (EtCO2 6-7 kPa) for another patient with severe acute respiratory distress syndrome to minimize lung injury.
- Oxygen saturation (SpO2) targets are also patient-specific. We might target SpO2 >94% for neuroprotection in one patient, but deliberately accept SpO2 >85% for a patient with cyanotic heart disease or SpO2 88-92 in a normal patient (Since the OXYPICU trial).
- Similarly, blood pressure and heart rate thresholds are highly age-dependent and further modulated by the presence of vasoactive infusions (as seen in 32.1% of our cohort), which profoundly alter expected "normal" ranges.

These individual, often case-specific, physiological targets mean that a single, fixed vital sign cutoff would frequently misclassify physiological stability as instability (false positives) or, more dangerously, miss true deterioration (false negatives). Fixed thresholds also cannot adapt to the gradual, clinically relevant changes in a patient's physiological baseline that occur throughout transport. As demonstrated by our previous work (Huo et al., 2023), vital signs exhibit dynamic trajectories; an initial fixed cutoff would quickly become unsuitable, resulting in either excessive false alarms or a failure to detect actual events as the patient's baseline shifts.

As we highlight in the Discussion section of our manuscript, "Traditional fixed-threshold alert systems... may therefore misclassify or overlook clinically relevant deviations in vital signs." Our method, by creating personalized and time-varying normal ranges, is specifically designed to overcome these limitations and adapt to the complex, individualized physiological trajectories of critically ill children, often by absorbing these gradual trends within the dynamic stable range to focus on acute changes.

Furthermore, it's important to reiterate that our study, did not have access to clinically verified "ground truth" labels for adverse events. As described in the Discussion, our attempts to use incident notes for this purpose revealed significant limitations. Consequently, we validated our labels against clinically meaningful surrogate markers (receipt of respiratory/vasoactive support and 30-day mortality). While a direct comparison to fixed cutoffs using these surrogates is theoretically possible, the fundamental clinical inappropriateness and variability of fixed thresholds across our diverse patient cohort mean such a comparison would likely yield limited insights into the true clinical utility of our personalized approach versus what is already a flawed comparator for this population.

Therefore, the potential benefit of our proposed approach lies not just in outperforming an arbitrary fixed threshold, but in providing a clinically relevant and adaptable method for identifying deviations from an individual's expected physiological state, a capability that fixed cutoffs inherently lack for critically ill, highly-managed patients. Our strong associations between the detected personalized events and important clinical outcomes serve as validation for the clinical meaningfulness of our labels.

To further strengthen our manuscript based on this valuable feedback, we have expanded the Discussion section to more explicitly detail the limitations of fixed vital sign thresholds in this patient population, including the specific clinical examples provided above and the issue of shifting baselines due to gradual trends, referencing our prior work (Huo et al., 2023). These revisions can be found in the second and new tenth paragraphs of the Discussion section.

- **Major Comment: “Introduction: Currently, the aim of the study isn't entirely clear from the Introduction: "we propose and validate a novel data-driven approach for automatically labelling patient-related adverse events, specifically respiratory and cardiovascular, using routinely collected vital signs data from the transport of critically unwell children." Could you also provide some insight into the way in which the proposed approach will be assessed, e.g. investigating the association between detected physiological adverse events and outcomes (namely treatment and mortality)?”**

*Response:* We appreciate the reviewer's comment regarding the clarity of our study's aim in the Introduction. We agree that explicitly stating how our proposed approach is assessed will enhance understanding.

We have now revised the final paragraph of the Introduction to clearly articulate our assessment strategy. We now explicitly state that we investigate the association between the detected physiological adverse events and key patient outcomes, including the receipt of respiratory and vasoactive support during transport, and 30-day mortality. This clarifies how we validate the clinical meaningfulness of our novel data-driven approach.

- **Major Comment: Conclusion - Rephrasing:**

**Sub-point (i) ""This approach reliably identified clinically significant events" - I don't think the results demonstrate that the detected physiological adverse events were clinically significant events. Rather, I think they demonstrated that "respiratory events and longer instability were significantly associated with increased receipt of respiratory support and higher 30-day mortality, while more cardiovascular events and longer instability correlated with greater receipt of vasoactive support". In my view, this is a little difference to identifying "clinically significant events", because "clinically significant events" suggests to me an event that requires a response, rather than a change that is associated with a future outcome. Perhaps I have misinterpreted this - in which case perhaps clarification would be helpful.":**

*Response:* We agree with the reviewer's interpretation and appreciate the clarification. We have rephrased the conclusion to be more precise and directly supported by our results. Instead of 'reliably identified clinically significant events,' we now state: 'Evaluation of our approach demonstrated that respiratory events and longer instability were significantly associated with increased receipt of respiratory support and higher 30-day mortality, while more cardiovascular events and longer instability correlated with greater receipt of vasoactive support. ' We have removed the phrase 'clinically significant events' to avoid misinterpretation and ensure the conclusion is directly tied to the evidence presented. We have also updated the concluding paragraph of the abstract to reflect this modified conclusion.

**Sub-point (ii) "The resulting labelled datasets pave the way for predictive AI models" - I don't think there is evidence in the results to support this statement (or at least in the way it is currently phrased), and I would suggest either omitting or at least re-phrasing it.":**

*Response:* We acknowledge the reviewer's concern regarding the phrasing of this statement. While our work aims to provide a foundation for future predictive models, we agree that the current results do not directly demonstrate this. We have revised or removed this statement to avoid overstating the current findings. "We have rephrased this to: The approach is readily adaptable to other high-resolution intensive care datasets, for both retrospective labelling as well as automated, real-time identification of adverse events in the clinical setting, offering a foundation for improved monitoring and early intervention in critically ill patients.

**Minor Comments:**

- **Methods > “Data Sources: Please could you state what sensing modalities were used to measure each vital sign? For instance, the manuscript currently states "vital signs, including heart rate, respiratory rate, blood pressure, oxygen saturation (SpO2), and end-tidal carbon dioxide (EtCO2)". However, at this point the reader is not aware of whether BP was invasive or non-invasive; whether heart rate was derived from ECG or pulse oximetry (or whichever is available); and the sensor used to obtain respiratory rate (e.g. impedance pneumography from ECG leads, or EtCO2 monitoring).”:**

*Response:* We thank the reviewer for this important clarification. We have added details on the sensing modalities used for each vital sign in the 'Data Sources' section of the Methods. Heart rate was derived from ECG, respiratory rate from both impedance pneumography and EtCO2, blood pressure was measured both non-non-invasively and invasively.

- **Methods > Data Sources: Similarly, this same sentence states "high-resolution (one data point per second) vital signs, including ... blood pressure". But surely when non-invasive BP is measured it doesn't provide new measurements each second. Perhaps cuff-based measurements are held at their previous value until the next cuff measurement? Please clarify if and how non-invasive BP measurements are measured at one data point per second.:**

*Response:* We appreciate the reviewer's observation regarding the sampling rate for non-invasive blood pressure. We have clarified in the 'Data Sources' section that while the monitoring system collects data at one-second intervals, non-invasive BP measurements are indeed cuff-based and are held at their last measured value until a new measurement is obtained.

- **Methods > Inclusion Criteria: "had at least 30 minutes of vital sign data". Please state somewhere in the manuscript (not necessarily here), how long it took to learn a patient-specific baseline (e.g. 15 mins, from EMA span of 15), and also, which period of time predictions of physiological adverse events were made on. For instance, if it does take 15 minutes to establish a baseline, then perhaps predictions are only made after the first 15 minutes of recording?**

*Response:* We thank the reviewer for raising this excellent point. We have added a clarification in the 'Construction of Bollinger Bands Section'. To clarify: for both respiratory and cardiovascular vital signs, an EMA with a Span of 15 was chosen. Therefore, at the start of each transport episode, 15 minutes of data was required to “warm-up” and calibrate the bands to the patients baseline. Consequently, adverse events were only labelled and subsequently analysed for periods following this initial 15-minute warm-up phase.

- **Methods > Adverse Respiratory Events: "identified using four parameters: (1) SpO₂ (plethysmograph), (2) plethysmograph-derived respiratory rate". This gave me the impression that both SpO2 and respiratory rate were obtained from the same sensor (called a plethysmograph here, which presumably refers to either photoplethysmography used in pulse oximeters, or a respiratory belt using inductance plethysmography or similar). Please make it clear (as per my earlier comment) which sensor(s) are used for which vital signs.**

*Response:* Thank you for highlighting the imprecise terminology regarding the respiratory rate measurement. You are entirely correct; our use of 'plethysmograph-derived respiratory rate' was indeed inaccurate and potentially misleading.

We have re-verified our data acquisition methods. The respiratory rate parameter referenced as 'plethysmograph-derived respiratory rate' was, in fact, obtained from impedance pneumography, which utilizes the same ECG electrodes for measurement on the Philips IntelliVue MP5 monitor.

We've now corrected the manuscript in both the 'Data Sources' and 'Adverse Respiratory Events' sections to accurately reflect that this respiratory rate is derived from impedance pneumography, ensuring clarity and avoiding any confusion with photoplethysmography from the SpO2 sensor

- **Results > Table 1: Please define "PIM3" in the caption:**

*Response:* "We have added a definition for 'PIM3' (Paediatric Index of Mortality 3) to the caption of Table 1."

- **Results > Labelling: "Using this technique, we identified at least one adverse respiratory event..." Please state the technique, as I don't think it's entirely clear which technique "this technique" refers to in this sentence.:**

*Response:* "We agree that the reference to 'this technique' was ambiguous. We have revised the sentence in the 'Labelling' section to explicitly state the technique being referred to. It now reads as follows: 'Using our personalized, data-driven approach for identifying deviations from individualized normal ranges, we identified at least one adverse respiratory event...'."

- **Results > Figure 2: appears pixelated in my PDF version. Please ensure the figures are clear.**

*Response:* "We apologize for the pixelation issue with Figure 2. We have re-uploaded a higher-resolution version of Figure 2 and Figure 3 to ensure clarity."

- **Acknowledgements: Please provide further details of the support from Kinseed.**

*Response:* "We have updated the Acknowledgements section to provide further details regarding the nature of the support received from Kinseed. Kinseed engineered the bespoke SwiftCare system for the real-time extraction and secure server upload of high-frequency vital sign data directly from patient monitors during transport.

### **List of Changes Made to the Manuscript**

- **Clarified the ethical restrictions and access procedures for data** as outlined in the Data Availability Statement.
- **Expanded the Discussion** to address the potential differences in vital sign profiles and the relevance of our approach for transported critically ill children compared to emergency presentations.
- **Included a statement in the Discussion** on the importance of studying gradual vital sign trends and its potential as a future research direction.
- **Detailed the rationale for not using fixed vital sign cutoffs as a comparator** in the Discussion, including specific clinical examples and addressing the challenges of individualized targets and shifting baselines.
- **Revised the final paragraph of the Introduction** to more clearly state the study's aim, specifically clarifying that we investigate the association between detected adverse events and key patient outcomes (respiratory/vasoactive support and 30-day mortality).
- **Rephrased the concluding statements in the Abstract and Conclusion sections** to more precisely reflect the study's findings regarding the association of events with clinical outcomes, rather than broadly stating "clinically significant events" or direct support for future predictive AI models.
- **Provided explicit details on the sensing modalities for each vital sign** in the Methods > Data Sources section (e.g., heart rate from 3-lead ECG, respiratory rate from impedance pneumography and EtCO2, blood pressure as invasive/non-invasive).
- **Clarified how non-invasive blood pressure data were handled** in the Methods > Data Sources section, explaining that cuff-based measurements were carried forward between readings.
- **Added a clarification in the Methods > Construction of Bollinger Bands section** detailing the 15-minute "warm-up" period required for EMA calibration, and specifying that adverse events were only labelled after this initial phase.
- **Corrected the terminology for respiratory rate derivation in the Methods > Adverse Respiratory Events section** (and Data Sources), changing "plethysmograph-derived respiratory rate" to "respiratory rate derived from impedance pneumography."
- **Defined "PIM3" (Paediatric Index of Mortality 3)** in the caption of Table 1.
- **Clarified the "technique" reference** in the "Labelling" section of the Results to explicitly refer to "our personalized, data-driven approach for identifying deviations from individualized normal ranges."
- **Re-uploaded higher-resolution versions of Figure 2 and Figure 3** to address pixelation concerns.
- **Expanded the Acknowledgements section** to provide specific details on Kinseed's contribution in developing the SwiftCare system for real-time, high-frequency vital sign data extraction and secure upload.

We believe that these revisions have substantially improved the manuscript and addressed all concerns raised. We are grateful for your time and consideration.

Sincerely,

Dr Milan Kapur, UCL Great Ormond Street Institute of Child Health
